# Medicines availability for non-communicable diseases: the case for standardized monitoring

**DOI:** 10.1186/s12992-015-0105-0

**Published:** 2015-05-07

**Authors:** Jane Robertson, Cécile Macé, Gilles Forte, Kees de Joncheere, David Beran

**Affiliations:** World Health Organization, 20, avenue Appia, 1211 Geneva, Switzerland; University of Newcastle, Calvary Mater Hospital, Newcastle, 2298 Australia; Geneva University Hospitals (HUG), 4, rue Gabrielle-Perret-Gentil, 1211 Geneva, Switzerland; University of Geneva, 24 rue du Général-Dufour, 1211 Geneva, Switzerland

**Keywords:** Essential medicines, Non-communicable diseases, Availability, Monitoring

## Abstract

**Background:**

In response to the global burden of non-communicable diseases (NCDs), the World Health Organization (WHO) has developed a Global Action Plan that includes a voluntary medicines target of 80% availability and affordability of essential medicines for the prevention and treatment of diabetes, cardiovascular disease and respiratory disease both in public and private health facilities. Reliable measures of medicines availability are needed to track progress towards meeting this target. The results of three studies measuring the availability of medicines for hypertension and diabetes conducted in Tanzania in 2012–2013 were compared to assess the consistency of the results across the studies.

**Methods:**

Availability was defined by observation of the medicine (no minimum quantity) on the day of the survey. The three studies involved 24, 107 and 1297 health facilities. Estimates of the availability of medicines for hypertension and diabetes were compared for medicines availability overall, by managing authority (government, mission/faith-based, private-for-profit), by facility level (hospital, health centre, dispensary) and by setting (urban, rural).

**Results:**

Comparisons of the availability of medicines were limited by differences in the definitions of the medicines and the classifications of the facilities surveyed. Metformin was variously reported as available in 33%, 39%, 46%, and 57% of facilities. Glibenclamide availability ranged from 19% to 52%. One study reported low levels of insulin availability (9-16% depending on insulin type) compared to 34% in a second study. Captopril (or angiotensin converting enzyme [ACE] inhibitor) availability ranged from 13% to 48%while availability of calcium channel blockers was 29% to 57% and beta-blockers 15% to 50%. Trends were similar across studies with lower availability in government compared to mission or private facilities, in dispensary and health centres compared to hospitals, and in rural compared to urban facilities.

**Conclusions:**

All three studies showed suboptimal availability of NCD medicines, however the estimates of availability differed. Regular monitoring using reproducible methods and measuring key medicines must replace ad-hoc studies, small selected samples and differences in definitions. Low and middle-income countries need to implement monitoring and evaluation systems to track progress towards meeting the NCD medicines target and to inform country-level interventions to improve access to NCD medicines.

## Background

Universal access to health care is heavily dependent on access to affordable essential medicines and health products [1]. Quality-assured essential medicines should be available at all times in adequate quantities, and at a price that both individuals and the community can afford [2]. Studies have repeatedly documented the low availability, high prices and poor affordability of key essential medicines for non-communicable diseases (NCDs) in many low- and middle-income countries (LMICs) both in the public and private sectors [3-6].

As the burden of illness in these settings now includes communicable and NCDs, there is renewed attention on the poor access to essential medicines for these conditions. Concern with the high morbidity and mortality associated with cardiovascular disease, cancer, chronic respiratory diseases and diabetes is reflected in the United Nations (UN) Political Declaration on NCDs which states that improving health systems and access to affordable medicines, particularly at the primary care level, is critical for their prevention and control [7]. As part of the global response, the World Health Organization (WHO) has developed a Global Action Plan and monitoring framework to enable tracking of progress in preventing and controlling these major NCDs [8]. The framework includes a voluntary medicines target of ‘an 80% availability of the affordable basic technologies and essential medicines, including generics, required to treat major non-communicable diseases in both public and private facilities’ [9].

A WHO Package of Essential Noncommunicable (PEN) disease interventions is designed to integrate the management of diabetes, cardiovascular and respiratory disease into primary health care [10]. The PEN program includes a list of recommended medicines that should be available in all primary health care facilities, recognising that although treatment may be initiated at higher levels of the health care system, patients are likely to use primary health care facilities for disease management and to access prescribed medicines. Reviews of facility surveys conducted in LMICs have shown that availability of generic medicines to treat NCDs is lower than for communicable diseases in both the public (36.0% vs. 53.5%) and private sectors (54.7% vs. 66.2%) [3].

Measuring progress towards achieving the 80% medicines target requires regular measurement of the availability of key NCD medicines, although there is currently no standardized methodology recommended by WHO for doing this. Few LMICs have country-level surveillance and monitoring systems in place to be able to chart progress, relying instead on ad-hoc studies and surveys to fill these information gaps. The reliability and representativeness of these ad-hoc survey data are often assumed. However, if availability estimates from the studies are similar, this would lend support to using the simplest, most efficient method of data collection in settings where routine data collection and monitoring systems are not yet in place. Tanzania was the only example we found where there was more than one study using different methods and with sufficient data on the availability of NCD medicines to allow meaningful comparisons. Data from three studies conducted in Tanzania in 2012–2013 [11-13] providing estimates of the availability of key essential medicines used to treat hypertension and diabetes allowed us to examine the consistency of the results across the studies and to compare the resulting inferences on access to essential medicines for these NCDs.

## Methods

Point-in-time estimates of the availability of medicines, based on the observation of the study medicines on the day of the visit regardless of the quantity of stock present, were extracted from each of the three studies.

Peck et al. assessed health facility preparedness for the outpatient treatment of hypertension and diabetes in Tanzania [11]. The assessment, conducted between November 2012 and May 2013, involved 24 public or private not-for-profit health facilities in the selected study areas, including four hospitals (two referral and two district hospitals), eight health centres (two urban and six rural) and 12 dispensaries (six urban and six rural). Purposive sampling was used to select hospitals for the study while other facilities were randomly selected. Medicines included in this study were metformin and short-acting, intermediate-acting, and long-acting insulins for the treatment of diabetes, and atenolol or propranolol, captopril or lisinopril, nifedipine, and hydrochlorothiazide or bendrofluazide for the management of hypertension.

A study using the World Health Organization-Health Action International (WHO-HAI) survey methodology was conducted in Tanzania in September 2012 [12]. The 107 health facilities surveyed included 37 Government (public) facilities, 34 Mission (Faith-based organization) facilities and 36 private-for-profit facilities (licensed pharmacies and drug stores). For each of the study survey areas, a minimum of five public sector outlets were chosen including the main public hospital and four medicine outlets (primary health care centres or district hospitals). Private-for-profit and mission medicine outlets are the closest facility of that type to the selected public sector facility. As a medicines prices and availability survey, the WHO-HAI study specifies the form and strength of the medicine surveyed. Relevant for this study were the following medicines: glibenclamide 5 mg and metformin 500 mg tablets for diabetes and atenolol 50 mg, captopril 25 mg and nifedipine retard 20 mg tablets for hypertension.

The third study was a World Health Organization Service Availability and Readiness Assessment (SARA) [13,14]. The assessment of service readiness reflects the ability of the facility to provide general and specific services at minimum standards and includes the availability of key medicines and health commodities. The sample of facilities is drawn from a master list of all public, private non-profit, private for-profit and faith-based health facilities, including hospitals, health centres, dispensaries and specialized clinics in the country. The SARA was conducted in 27 districts in Tanzania with a final sample of 1297 facilities, representing more than 18% of all health facilities in Tanzania and stratified by facility level (dispensary, health centre, hospital), managing authority Government/public, Mission or Faith-based organization, not-for-profit NGO, private-for-profit), ownership (Government/public, private) and residence (urban and rural). Medicines data were obtained from general service assessments of all 1297 health facilities surveyed (specific tracer medicines: glibenclamide 5 mg, atenolol 50 mg, captopril 25 mg tablets) and from facilities meeting standards for providing diabetes services (248 facilities: glibenclamide, metformin, insulin) and cardiovascular services (316 facilities: beta-blockers, ACE inhibitors, calcium channel blockers, thiazide diuretics).

In order to compare the results from the different designs, the analyses presented show the mean availability (%) for the medicines across the facilities surveyed and as reported in each of the three studies. Data were extracted by two authors working independently with discrepancies managed by discussion and consensus. In addition, where possible, data were re-analysed using the raw data to allow comparisons of medicines availability overall and by facility setting that were not presented in the original publications. To examine distributional effects, the mean availability of metformin and ACE inhibitors (recommended first line treatments in PEN guidelines for diabetes and hypertension respectively) is summarised by managing authority (government, mission or faith-based organization, private-for-profit), by facility level (hospital, health centre, dispensary) and by setting (urban, rural).

Ethics approval was not required for this study that uses aggregate data from publicly available data sources.

## Results

The differences in the level of specification of the medicines included in the three studies and the classification of the health facilities surveyed affected the comparisons on medicines availability that could be made (Table 1).

**Table 1:** **Availability of medicines for diabetes and hypertension**

**Medicine**	**Mean availability (%) of medicine**				
	**Peck et al.**	**WHO/HAI**	**SARA**		
			**All facilities#**	**Diabetes services**	**Cardiovascular disease services**
	**N = 24**	**N = 107**	**N = 1297**	**N = 248**	**N = 316**
**Diabetes**					
Glibenclamide		47*	19*	52	
Metformin	33	46†		57	39
Insulin	17; 8; 8‡			34	
**Hypertension**					
Captopril 25 mg		48	13		
Captopril or lisinopril	25				
ACE inhibitors					24
Nifedipine	33	57§			
Calcium channel blockers					29
Atenolol 50 mg		48	15		
Atenolol or propranolol	50				
Beta-blockers					41
Hydrochlorothiazide or bendrofluazide	33				31¥

FBO = Faith Based Organization; SR = sustained release formulation.

#Availability of essential tracer medicines is measured across all health facilities surveyed.

*glibenclamide 5 mg tablet.

†metformin 500 mg tablet.

‡short-acting, intermediate-acting and long-acting insulin assessed separately.

§WHO/HAI survey specified 20 mg sustained-release formulation.

¥specified as thiazide diuretics.

### Diabetes medicines

Glibenclamide availability ranged from 19% (5 mg tablet, all facilities SARA survey) to 52% (no strength specified, SARA diabetes services). Metformin 500 mg was available in 46% of facilities in the WHO-HAI survey, metformin availability (no strength specified) ranged from 33% (Peck et al.) to 57% (SARA diabetes services). Metformin was also reported available in 39% of facilities providing cardiovascular services (SARA). Peck et al. reported low levels of availability of insulin (8-17% depending on insulin type) while at least one type of insulin was available in 34% of facilities providing diabetes services (SARA). Insulin availability was not examined in the WHO-HAI survey.

### Hypertension medicines

The reported availability of captopril 25 mg tablets ranged from 13% (SARA all facilities) to 48% (WHO-HAI survey). There was only 24% availability reported for the broader category ‘ACE inhibitors’ in facilities providing cardiovascular services (SARA). Availability of calcium channel blockers ranged from 29% (no specified medicine SARA cardiovascular services) to 57% (nifedipine SR 20 mg WHO-HAI survey). Availability of atenolol 50 mg tablets was reported in 48% of facilities in the WHO-HAI survey however in only 15% of all facilities in the SARA. Peck et al. reported 50% availability of atenolol or propranolol in the 24 facilities surveyed. Estimates of the availability for thiazide diuretics were similar in SARA cardiovascular services (31%) and the Peck et al. study (33%).

### Analyses by managing authority, facility level and setting

Availability of metformin was lower in government compared to mission or private facilities in both the WHO-HAI survey and the SARA, although the numeric estimates differed considerably between the studies (30%, 65%, 44% compared to 42%, 93%, 82%, Table 2). Likewise, there was a consistent pattern of greater availability of metformin in hospital than in health centre and dispensary settings, and in urban compared to rural settings. However, there were substantial differences in these estimates between the Peck et al. and SARA diabetes services estimates (Table 2). Notably, Peck et al. reported no availability of metformin in rural facilities surveyed while availability was 42% in the SARA.

**Table 2:** **Availability of metformin by facility type and setting**

**Medicine**	**Observed mean availability (%) of medicine**		
	**Peck et al.**	**WHO/HAI survey**	**SARA**
			**Diabetes services**
	**N = 24**	**N = 107**	**N = 248**
	Metformin*	**Metformin 500 mg**	**Metformin**
**Metformin**			
Government facility		30	42
Mission/Faith-based organization		65	93
Private facility		44	82
Hospital	100		87
Health centre	13		65
Dispensary	25		48
Urban	75		75
Rural	0		42

*Metformin 500 mg is listed in 2013 Tanzania National Essential Medicines List.

Similar patterns of availability applied to ACE inhibitors, notwithstanding the differences in level of specification of the medicines in each study (Table 3). As with metformin, there were notable differences in the estimates of availability between studies.

**Table 3:** **Availability of ACE inhibitors by facility type and setting**

**Medicine**	**Observed mean availability (%) of medicine**		
	**Peck et al.**	**WHO/HAI survey**	**SARA**
	**N = 24**	**N = 107**	**Cardiovascular services (N = 316)**
	***Captopril or lisinopril***	***Captopril 25 mg***	ACE inhibitors*
**ACE inhibitors**			
Government facility		30	17
Mission/Faith-based organization		59	42
Private		56	39
Hospital	25		67
Health centre	13		30
Dispensary	33		16
Urban	50		36
Rural	0		17

*Captopril and perindopril listed in 2013 Tanzania National Essential Medicines List.

## Discussion

The three studies were conducted in a similar time period yet provide substantially different estimates of the availability of medicines for the treatment of diabetes and hypertension in Tanzania.

The overall conclusions of each of the three studies are consistent, i.e. that availability of key NCD medicines for the management of diabetes and hypertension is sub-optimal. In addition, there were consistent patterns of lower availability in government facilities than mission/faith-based and private facilities with lower availability in dispensaries and health centres than in hospitals and lower availability in rural than urban health facilities. These differences will impact on the ability to provide equitable access to diabetes and hypertension treatments for patients. However estimating the extent of these problems is difficult given the ranges of estimates in the three studies.

The abstract of the Peck et al. study states that “a representative sample of 24 public and not-for-profit health facilities in urban and rural Tanzania” was used. These authors refer to the use of an adapted version of the WHO SARA questionnaire to assess facility readiness to provide chronic disease services. However the sample size used is much smaller than that used in a nationally representative SARA. A WHO-HAI survey provides point in time estimates of medicines availability (and prices). However it uses purposive rather than representative sampling, aiming to strike a balance between representativeness and practicality [15]. The limited stratification of health facilities in WHO-HAI surveys will limit the usefulness of the data for sector planning within countries.

SARAs are carried out at the request of Ministries of Health in advance of planned country health policy reviews in order to inform health decision-making. Sampling methods used depend on country application. A nationally representative random sample of at least 150 health facilities can be used to obtain national estimates, while a district-level assessment with a census of all facilities in selected districts can generate results that can be used for local management [14]. The SARA for Tanzania included more than 18% of all health facilities in Tanzania, and will therefore most closely reflect the situation of medicines availability in the country. With analyses stratified by facility type, operating authority, ownership and residence (urban and rural areas) a more detailed understanding of within-country problems of medicines availability is possible, allowing targeted interventions to address procurement and distribution issues. However a SARA is a resource intensive data collection exercise and to date, these have mostly been conducted in Africa with the support of donors.

The availability and affordability of medicines are central to health service delivery in any community. When medicines are not available in the public sector people go elsewhere for their health care, often forced into the private sector where medicines are more available but also more expensive, indeed unaffordable for some. Increasing access to essential, quality-assured, safe, effective and affordable medical products is one of the global leadership priorities for WHO [16]. The results presented here relate to medicines availability, only the WHO-HAI study also assessed medicines prices.

As the disease burden in LMICs shifts from communicable to NCDs, the issue of the availability and affordability of critical NCD medicines becomes more important. NCDs represent a paradigm shift in the way health systems are organized and in treatment approaches as patients need to understand that treatment does not stop once they feel better and that life-long therapy is required for sometimes asymptomatic conditions like hypertension and hyperlipidaemia. No medicines or interrupted medicines supplies in public facilities, difficulties in accessing medicines from other sources and higher than necessary costs in the private sector will compromise the adherence to treatment needed to achieve the desired clinical outcomes.

The results presented here suggest problems with availability of NCD medicines, particularly in the public sector and rural areas, leading to low availability of NCD medicines that are themselves relatively cheap. Medicines like metformin, glibenclamide and ACE inhibitors are long out of patent, and there are multi-source products. Availability and affordability of insulin is a well-recognised problem in low and middle-income countries, with the additional costs of syringes and other diabetes commodities adding to the treatment burden [17].

None of the three studies explored reasons for the low availability of medicines reported. The estimates should stimulate further enquiry as to why the medicines are not available. For example, lower availability in the public sector across all facility types and locations compared to the private sector could reflect inadequate government funds to purchase sufficient medicines to meet patient needs. If availability in the public sector is consistently lower in rural areas, it could suggest problems with procurement and distribution systems to the more distant health facilities. Paradoxically, medicines being available could mean that the medicines are not being prescribed by the health care providers in that district (i.e. low demand). Different types of follow-up enquiry are required in each case to determine the causes of the poor availability.

These availability surveys provide no information on the diagnosis and management of hypertension and diabetes or the treatment choices of health care professionals. Recent estimates suggest that many individuals with hypertension in Africa are unaware of their condition [18]. In addition, hypertension may rank low as a health priority competing with treatments for infectious diseases for limited resources [19]. Low demand may also explain in part the relatively low availability in the private sector reported here.

With the global focus on improvements in access and affordability of NCD medicines, it is essential to have a reliable platform for measuring supply system weaknesses and assessing changes in medicines availability. A fragmented approach to measurement relying on ad-hoc studies leads to information gaps and duplication of efforts while limiting the ability to monitor trends over time. Preferable is systematic monitoring of medicines availability and prices that can help identify potential problems and the corrective actions required and a culture of using data to inform decision-making and planning at the country level. LMICs will require support to develop these monitoring systems.

The World Health Assembly (WHA) 2013 focused on the linkages of NCDs to Universal Health Coverage (UHC) and the role of essential medicines in improving patient access to affordable medicines [20]. The measurement of NCD medicines availability is not only relevant to assessing the 80% medicines availability target within the NCD Global Action Plan, but for the wider achievement of UHC. This monitoring should be built into public health programs at the national, regional, and service delivery level and requires routine collection of good quality data that are valid and reliable, using standardised and reproducible methods. Methods for data collection ideally should be simple, focus on a smaller number of key medicines, and be able to be undertaken on a regular basis, preferably without substantial additional costs to the health services. Monitoring, evaluation and review of data with a view to corrective actions should be part of the national health strategy.

## Conclusions

Given the differences between study estimates, none of the three methods completely meets the needs for monitoring availability of NCD medicines at a country level. The challenge is establishing a meaningful platform from which to judge the effects of efforts to improve access to medicines. There is an urgent need for a different approach that moves beyond ad-hoc studies and focuses on reliable and reproducible information to support decision-making. Countries need to build this into their routine information systems. Along with this, there must be a culture of using information to investigate problems and propose concrete solutions to improve access to essential medicines.

## Abbreviations

ACE: Angiotensin converting enzyme
FBO: Faith based organization
LMIC: Low and middle-income country
NCD: Non-communicable disease
NGO: Non-government organization
PEN: Package of Essential Non-communicable disease
SARA: Service availability and readiness assessment
SR: Sustained release
UHC: Universal health coverage
WHA: World Health Assembly
WHO-HAI: World Health Organization-Health Action International

## References

[1] World Health Organization. Key components of a well-functioning health system. Geneva: World Health Organization, 2010. [http://www.who.int/healthsystems/publications/hss_key/en/]

[2] World Health Organization. The Selection and Use of Essential Medicines. Report of the WHO Expert Committee, 2013. Technical Report Series 985. [http://www.who.int/medicines/publications/essentialmeds_committeereports/en/]

[3] Cameron A, Roubos I, Ewen M, Mantel-Teeuwisse AK, Leufkens HG, Laing RO (2011). Differences in the availability of medicines for chronic and acute conditions in the public and private sectors of developing countries. Bull World Health Organ.

[4] Mendis S, Fukino K, Cameron A, Laing R, Filipe A, Khatib O (2007). The availability and affordability of selected essential medicines for chronic diseases in six low- and middle-income countries. Bull World Health Organ.

[5] Cameron A, Ewen M, Ross-Degnan D, Ball D, Laing R (2009). Medicine prices, availability, and affordability in 36 developing and middle-income countries: a secondary analysis. Lancet.

[6] van Mourik MS, Cameron A, Ewen M, Laing RO (2010). Availability, price and affordability of cardiovascular medicines: a comparison across 36 countries using WHO/HAI data. BMC Cardiovasc Disord.

[7] UN General Assembly (2012). Political Declaration of the High-level Meeting of the General Assembly on the Prevention and Control of Non-communicable Diseases.

[8] World Health Organization. Global Action Plan for the Prevention and Control of Noncommunicable Diseases 2013–2020. Geneva: World Health Organization, 2013. [http://www.who.int/nmh/events/ncd_action_plan/en/]

[9] World Health Organization. NCD Global Monitoring Framework: ensuring progress on noncommunicable diseases in countries. [http://www.who.int/nmh/global_monitoring_framework/en/]

[10] World Health Organization. Implementation tools Package of Essential Noncommunicable (PEN) disease interventions for primary health care in low-resource settings. Geneva: World Health Organization, 2013. [http://www.who.int/cardiovascular_diseases/publications/implementation_tools_WHO_PEN/en/]

[11] Peck R, Mghamba J, Vanobberghen F, Kavishe B, Rugarabamu V, Smeeth L (2014). Preparedness of Tanzanian health facilities for outpatient primary care of hypertension and diabetes: a cross-sectional survey. Lancet Global Health.

[12] WHO/HAI. Measuring medicine prices, availability, affordability and price components. 2nd Edition. Geneva, Switzerland; 2008 [http://www.haiweb.org/medicineprices/manual/documents.html]

[13] Ministry of Health and Social Welfare, Tanzania. Tanzania Service Availability and Readiness Assessment (2012). Final report July 2013. [http://www.ihi.or.tz/announ/availabilityandreadinessassessmentresultsannounced]

[14] O’Neill K, Takane M, Sheffel A, Abou-Zahr C, Boerma T (2013). Monitoring service delivery for universal health coverage: the Service Availability and Readiness Assessment. Bull World Health Organ.

[15] Madden JM, Meza E, Ewen M, Laing RO, Stephens P, Ross-Degnan D (2010). Measuring medicine prices in Peru: validation of key aspects of WHO/HAI survey methodology. Rev Panam Salud Publica.

[16] World Health Organization. WHO’s Leadership Priorities. [http://www.who.int/about/who_reform/change_at_who/leadership_priorities/en/#.VUp0xfmqqko]

[17] Beran D, Yudkin JS (2010). Looking beyond the issue of access to insulin: what is needed for proper diabetes care in resource poor settings. Diabetes Res Clin Pract.

[18] Adeloye D, Basquill C (2014). Estimating the prevalence and awareness rates of hypertension in Africa: a systematic analysis. PLoS ONE.

[19] Beaglehole R, Bonita R, Horton R, Adams C, Alleyne G, Asaria P (2011). Priority actions for the non-communicable disease crisis. Lancet.

[20] World Health Organization 2014. Access to Medicines. Sixty-seventh World Health Assembly WHA67.22. [http://apps.who.int/gb/ebwha/pdf_files/WHA67/A67_R22-en.pdf]

