# Erratum to: Medicines availability for non-communicable diseases: the case for standardized monitoring

**DOI:** 10.1186/s12992-015-0120-1

**Published:** 2015-08-06

**Authors:** Jane Robertson, Cécile Macé, Gilles Forte, Kees de Joncheere, David Beran

**Affiliations:** World Health Organization, 20 avenue Appia, Geneva, 1211 Switzerland; University of Newcastle, Calvary Mater Hospital, Newcastle, 2298 Australia; Geneva University Hospitals (HUG), 4, rue Gabrielle-Perret-Gentil, Geneva, 1211 Switzerland; University of Geneva, 24 rue du Général-Dufour, Geneva, 1211 Switzerland

## Erratum

The copyright line was incorrect in the published manuscript.

The copyright line should be:

*© 2015 World Health Organization; licensee BioMed Central.*

*This is an open access article distributed under the terms of the Creative Commons Attribution IGO License (**http://creativecommons.org/licenses/by/3.0/igo/legalcode**), which permits unrestricted use, distribution, and reproduction in any medium, provided the original work is properly cited. In any reproduction of this article there should not be any suggestion that WHO or this article endorse any specific organisation or products. The use of the WHO logo is not permitted. This notice should be preserved along with the article’s original URL*.

The disclaimer for WHO authors and non-WHO authors should be:

The authors alone are responsible for the views expressed in this article and they do not necessarily represent the views, decisions or policies of the institutions with which they are affiliated.

